# The influence of parenting style in childhood on adult depressed patients’ interpersonal relationships in the period of youth

**DOI:** 10.3389/fpsyg.2023.1169311

**Published:** 2023-07-31

**Authors:** Jingjing Chang, Kaiyi Huang, Weidong Wang

**Affiliations:** ^1^Guang’anmen Hospital, China Academy of Chinese Medical Sciences, Beijing, China; ^2^Qinhuangdao Beidaihe Hospital, Hebei, China

**Keywords:** depression, self, encourage, interpersonal relationships, parenting style

## Abstract

**Objective:**

The objective of this study was to explore the mediating effect of adolescent self and courage on the relationship between parenting style in childhood and adult depressed patients’ interpersonal relationships in the period of youth.

**Methods:**

The study analyzed data from 651 depressed individuals using the Wang Weidong memory-tracing personality developmental inventory (WMPI) from the psychology department of Guang’anmen Hospital.

**Results:**

The results of the study show a significant positive correlation between parenting style in childhood, adolescent self, courage, and adult depressed patients’ interpersonal relationships in the period of youth. Parenting style in childhood has a direct positive predictive effect on adult depressed patients’ interpersonal relationships in the period of youth. It also has an indirect effect on interpersonal relationships in the period of youth through three indirect pathways: the independent mediating effect of adolescent self, the independent mediating effect of adolescent courage, and the chain mediating effect of adolescent self and courage.

**Conclusion:**

The findings of this study suggest that parenting style in childhood plays an important role in shaping adult depressed patients’ interpersonal relationships in the period of youth. The relationship between parenting style in childhood and interpersonal relationships in the period of youth is influenced by the independent mediating effect of adolescent self and courage, as well as the chain mediating effect of adolescent self and courage. These findings have implications for the development of interventions and programs aimed at improving the mental health and well-being of depressed patients.

## Introduction

Depression is a mental disorder that is closely related to personality, and is mainly manifested as a symptom group of depression. Depression is a disease with a high prevalence and recurrence rate, which can easily trigger or aggravate other physical diseases and cause great damage to human health and property. The latest global burden of disease study shows that depression causes 1.85% of all disability-adjusted life years worldwide (GBD 2019 Diseases and Injuries Collaborators, 2020).

### Prevalence and consequences of depression

According to epidemiological data, the lifetime prevalence rate of depressive disorder in Chinese adults is 6.8%, with depression accounting for 3.4%. Among patients diagnosed with depressive disorder in the past 12 months, 75.9% have social dysfunction (Lu J. R. et al., 2021; Lu J. et al., 2021). Depression is significantly correlated with interpersonal relationships, and the level of interpersonal relationships of depressed patients changes to varying degrees (Liu, 2021).

### Potential factors favoring the depressive onset

Biologically, several genes have been shown to possibly increase the risk of depression (Wittenberg et al., 2020), and FMRI suggests that some volume, structure, and function changes in the brain may be closely related to depression (Amiri et al., 2021; Hopman et al., 2021). In addition, intestinal flora and metabolites and neuroimmune factors may also influence the development of depression (Stevens et al., 2021). Research has shown that the combined influence of family environment and parenting style on individual psychology can reach 60.8% (Liu and Jiang, 2018). Additionally, childhood parenting style has been found to easily lead to the development of depression (Zhang et al., 2019; Wen et al., 2022). Furthermore, Interpersonal therapists believe that interpersonal relationships constitute 80% of the causes of depression (Bandura et al., 1999), and the level of interpersonal relationships is an important factor affecting the depressive mood of adolescents (Wang et al., 2001; Dang et al., 2016). Poor interpersonal relationships play an important role in the initiation and maintenance of depressive mood (Rudolph et al., 2008), and are more likely to lead to non-suicidal self-injury behaviors among adolescents with depression (Xiang et al., 2022). Therefore, depressive conditions and interpersonal disorders often interact with each other.

### The link between depression and personality

Personality in Western psychology refers to the more stable traits of personality behavior and way of thinking. In the field of research on psychiatric disorders, personality traits are often key factors in the development of psychological problems, and there is no single personality element affecting depression. Studies have found that people with low self-direction and high harm avoidance are more likely to suffer from depression, while Chinese people have greater defensiveness and masking of depression susceptibility traits than Westerners, such as emphasizing their somatic symptoms and denying that the illness is related to their personality.

## Objectives and assumptions

Family is the smallest social structure experienced by individuals, and the parenting style of parents plays a crucial role in individual growth and development. Parenting style refers to the comprehensive embodiment of parents’ parenting concepts, behaviors, and emotions toward their children. It has a relatively stable behavior style and tendency that can have an impact across situations and time (Wang, 2014). Studies have shown that childhood is the basis and important stage of personality development in individual growth, and parenting styles and various dimensions have a direct and significant impact on the childhood personality development of patients with depression (Zhang, 2022).The parenting style of parents can influence the formation of future interpersonal relationship models. Individuals establish their initial interpersonal relationships under the parenting style of their parents, which can affect the formation of future interpersonal relationship models (Markiewicz et al., 2001). In traditional Chinese culture, there is an old saying, “Three years old and seven years old,” which affirms the important influence of early development on the future growth of individuals. Therefore, this study proposes Hypothesis 1: childhood parenting style has a direct predictive effect on adult depressed patients’ interpersonal relationships in the period of youth.

The personality system is an inseparable whole that includes various elements such as parenting style, self, courage, will, way of thinking, interpersonal relationship, temperament outlook, and world outlook. The growth of these elements is constantly changing, experiencing the formation, development, improvement, and end of the process. Therefore, the consideration of personality development should not be limited to a single, current time or space structure. The process of adaptation of infants to the natural environment and parenting style forms their basic courage. Around the age of 3, self-development occurs in conjunction with the development of courage. After the age of 3, courage becomes stronger with self-reinforcement, and together, they constitute the “core layer” of personality formation (Liu et al., 2019). As individuals grow older and expand their interpersonal interactions, they develop their courage and self in contradiction under the combined effect of various life experiences and parenting styles, and form basic interpersonal patterns. Research has shown that courage and self-play an important role in individual interpersonal communication (Tang et al., 2015). Mature and complete personality tends to have more harmonious and satisfying interpersonal relationships. Therefore, this study proposes Hypothesis 2: Childhood parenting style plays a role in adult depressed patients’ interpersonal relationships in the period of youth through adolescent self, and adolescent self is the mediating variable between childhood parenting style and the interpersonal relationships in the period of youth. Furthermore, Hypothesis 3 proposes that childhood parenting style indirectly affects adult depressed patients’ interpersonal relationships in the period of youth through adolescent self and courage, and adolescent self and courage play a chain mediating role.

## Data and methods

### Research subjects

In this study, WMPI scales were collected from 700 depressed patients, who were eligible for DSM-V diagnosis attending the psychology department of Guang'anmen Hospital from 2015 to 2022. The following exclusion criteria were applied: age ≤ 20 years old, age > 20 when sleeping apart from adults, polygraph score ≥ 18, answer time < 40 groups or > 90 min, and the absolute value of the retest difference of the juvenile version of the questionnaire was greater than or equal to 3. If a sample met any of the above criteria, it was excluded. The final effective scale included 651 samples, with an effective rate of 93%.

### Tool

Wang’s Retrospective Personality Development Scale (WMPI) is a personality assessment tool specific to China, which utilizes a retrospective approach to assess three stages of individual personality development and consists of 9 subscales. The total scale has a Cronbach’s Alpha coefficient of 0.990 and has been recognized by the Professional Committee of Psychometrics of Chinese Psychology Association. The subscale for childhood parenting style (from 3 years old to before primary school) consists of 29 items from 6 dimensions, including sternness, punishment, indulgence, excessive interference, contradictory education, and neglect of protection. The subscale employs a 5-point scoring system (0 = completely inconsistent, 4 = completely consistent), with a higher score indicating a higher degree of unreasonable parenting style. The subscale for childhood parenting style has a Cronbach’s Alpha coefficient of 0.887. Adolescent self includes 5 dimensions of social self, family self, physiological self, autonomy, and self-rationality, with a total of 20 items. The subscale has a Cronbach’s Alpha coefficient of 0.905. Adolescent courage includes 4 dimensions of interpersonal fear, natural fear, unknown fear, and adaptation, with a total of 29 items. The subscale has a Cronbach’s Alpha coefficient of 0.879, and a higher score indicates a weaker degree of courage. The subscale for adolescent interpersonal relationships includes three dimensions of agreeableness, altruism, and attachment, with a total of 18 items. The subscale has a Cronbach’s Alpha coefficient of 0.831, and a higher score indicates poorer interpersonal skills (Wang et al., 2016). The childhood, adolescence, and youth described in this study are the previous periods of the patients’ development, i.e., the periods of childhood, adolescence, and youth.

### Data processing

SPSS 26.0 was utilized to conduct descriptive, correlation, and regression analyses on the data. In addition, PROCESS was employed to test the mediation model. Model 6, provided by Hayes, was selected for analysis, and the non-parametric percentile Bootstrap method was used to assess the significance level of the mediation effect.

### Common method bias

The Harman single factor method was utilized to examine the common method bias of the study, and the first factor explained 18.05% of the variance, which was less than the critical value of 40%. This suggests that there is no significant common method bias present in the data of this study.

## Results

### Descriptive statistics

In this study, a total of 651 valid data were collected, including 181 males (27.80%) and 470 females (72.20%); the ages ranged from 21 to 75 years old; the education level of junior high school or below was 162, accounting for 24.88%, technical secondary school or high school was 170, accounting for 26.11%, college degree or above was 319, accounting for 49.00%; on the job was 585, accounting for 89.86%, off the job was 66, accounting for 10.44%; being married was 415, accounting for 63.75%, not being married was 236, accounting for 36.25%; the education level of main caregiver of junior high school or below was 275, accounting for 42.24%, technical secondary school or high school was 201, accounting for 30.88%, college degree or above was 175, accounting for 26.88%. The detailed results are shown in Table 1.

**Table 1:** General information.

Character		*N*	%
Gender	Male	181	27.80
	Female	470	72.20
Age	21–40	420	64.52
	41–60	182	28.00
	61–75	49	7.53
Educational level	Junior high school or below	162	24.88
	Technical secondary school or high school	170	26.11
	College degree or above	319	49.00
Occupation	On the job	585	89.86
	Off the job	66	10.14
Marital status	Being married	415	63.75
	Not being married	236	36.25
Education level of main caregiver	Junior high school or below	275	42.24
	Technical secondary school or high school	201	30.88
	College degree or above	175	26.88

### Correlation analysis between general information and relationships in the period of youth

The correlation between adult depressed patients’ interpersonal relationships in the period of youth and occupation, marriage, education level, and primary caregiver’s education level was tested, and the results showed that marriage, occupation and primary caregiver’s education level were correlated with interpersonal relationships in young adulthood (*p*< 0.05), with correlation coefficients of 0.240, 0.098 and − 0.125, respectively, while education level were not significantly correlated (*p* > 0.05). There is a positive correlation between interpersonal relationships and marriage, i.e., the more likely the patient is to be in a non-marital situation, the more pronounced the interpersonal impairment is. Some studies have shown that depressed individuals have significant interpersonal distrust and negative coping styles compared to the normal population. Individuals with high interpersonal mistrust are more likely to feel alienated and disconnected from others around them, and the stronger the depression, the more likely they are to adopt negative coping styles, which may contribute to the development of negative emotions into psychological problems or depression (Liu et al., 2019), and interpersonal mistrust and negative coping styles may contribute to non-marital status. Non-employees are more likely to have poor interpersonal relationships, possibly due to the loss of stable social contacts. The higher the primary caregiver’s educational level, the lower the interpersonal score, and the weaker the interpersonal impairment, probably because parents with higher education may be more likely to help their children develop good interpersonal skills.

### Correlation analysis among variables

According to the information presented in Table 2, a significant positive correlation exists among childhood parenting style, adolescent self, adolescent courage, and interpersonal relationships in depressed youth.

**Table 2:** Correlation analysis between variables.

	*M*	SD	1	2	3	4
1.Parenting style in childhood	38.482	18.413	1			
2. Adolescent self	34.430	10.307	0.266**	1		
3. Adolescent courage	56.863	15.301	0.289**	0.714**	1	
4.Relationships in youth	31.844	8.255	0.138**	0.623**	0.639**	1

**p < 0.01.

### Test of mediating effect

Table 3 illustrates that the SPSS macro program Process was employed to investigate the mediating effect. Specifically, the mediating effect of adolescent self and courage on the relationship between childhood parenting style and adult depressed patients’ interpersonal relationships in the period of youth was assessed while controlling for gender and age. Results of the regression analysis indicated that childhood parenting style had a direct positive predictive effect on both adolescent self (*β* = 0.148, *p* < 0.001) and courage (*β* = 0.104, p < 0.001), while adolescent self had a direct positive predictive effect on courage (*β* = 1.048, p < 0.001). When childhood parenting style, adolescent self, and courage were all used to predict adult depressed patients’ interpersonal relationships in the period of youth, they all had a significant positive effect on interpersonal relationships (*β* = 0.031, *p* < 0.001; *β* = 0.280, *p* < 0.001; *β* = 0.214, *p* < 0.001).

**Table 3:** Regression analysis between variables.

Regression equation		Overall fit index			Significance of regression coefficient		
Result variable	Predictive variable	*r*	*r*	*F*	*β*	*SE*	*t*
S-self		0.30	0.09	21.00**			
	Gender				−2.78	0.88	−3.16**
	Age				−0.02	0.03	−0.78**
	T-parenting style				0.15	0.02	7.03**
S-courage		0.75	0.56	205.68**			
	Gender				4.25	0.92	4.64**
	Age				−0.07	0.03	−2.27*
	T-parenting style				0.10	0.02	4.61**
	S-self				1.05	0.04	25.83**
Q-relationship		0.70	0.48	120.99**			
	Gender				0.51	0.54	0.94
	Age				−0.09	0.02	−4.57**
	T-parenting style				0.03	0.01	2.32*
	S-self				0.28	0.03	8.27**
	S-courage				0.21	0.02	9.29**

**p < 0.01, *p < 0.05.

**Figure 1:**
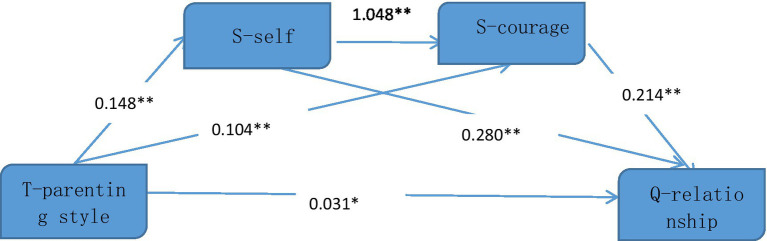
Chain mediation diagram. **p < 0.01, *p < 0.05.

Table 4 demonstrates that the non-parametric percentile Bootstrap method with deviation calibration is utilized to assess the significance level of the mediating effect. Results indicate that the mediating effect of adolescent self and courage is significant, with a mediating effect value of 0.033. The mediating effect is generated through three mediating chains. Firstly, the indirect effect composed of childhood parenting style → adolescent self → interpersonal relationship is 0.041. The Bootstrap 95% interval does not contain 0, suggesting that the mediating effect of adolescent self is significant. Secondly, the indirect effect of childhood parenting style → adolescent self → courage → interpersonal relationship is 0.033. The Bootstrap 95% interval does not contain 0, indicating that adolescent self and courage have significant chain mediating effects on childhood parenting style and interpersonal relationship in depressed youth. Thirdly, the indirect effect of childhood parenting style → courage → interpersonal relationship is 0.022, and the Bootstrap 95% interval does not contain 0, indicating the significant mediating effect of adolescent courage. The specific path of the effect of childhood parenting style on adult depressed patients’ interpersonal relationships in the period of youth is illustrated in the Figure 1.

**Table 4:** The mediating effect of S-self and S-courage on T-parenting style and Q-interpersonal relationship.

Path	Effect value	Bootstrap	Bootstrap 95% interval		Relative mediation Effect ratio
		SE	BootLLCL	BootULCL	
Total indirect effect	0.097	0.012	0.074	0.122	
T-parenting style →S-self →Q-interpersonal relationship	0.041	0.008	0.027	0.059	42.27%
T-parenting style →S-self →S-courage →Q-interpersonal relationship	0.033	0.006	0.022	0.046	34.02%
T-parenting style →S-courage →Q-interpersonal relationship	0.022	0.006	0.012	0.034	22.68%

**p < 0.01, *p < 0.05.

## Discussion

The findings of this study demonstrate a significant positive correlation between childhood parenting style and adolescent self, courage, and adult depressed patients’ interpersonal relationships in the period of youth. This suggests that childhood parenting style can impact an individual’s self, courage, and interpersonal relationships during adolescence, while the interpersonal relationships in the period of youth is also influenced by childhood parenting style, adolescent self, and courage. Overall, the study highlights the importance of parenting style in shaping the developmental outcomes of adolescents, particularly in relation to their mental health and well-being.

Human development and growth is a complex process that involves multiple factors, including past experiences and current circumstances. Prior research has demonstrated that parenting style has continuity characteristics, and childhood parenting style can have a significant impact on adolescent and youth development. Additionally, parenting style can also influence an individual’s self and self-concept in subsequent periods of their lives. The findings of this study support previous research that has highlighted the important role of childhood parenting style in shaping adolescent self-esteem and resilience. A more reasonable and supportive childhood upbringing can promote greater adaptability, security, and confidence during adolescence, which can have positive effects on an individual’s social relationships and overall well-being. Erikson’s concept of self-identity is also relevant to understanding the developmental outcomes of adolescents and youth. Adolescents who have a stronger sense of self-identity are more likely to have successful social interactions and develop meaningful relationships with others. These positive outcomes during adolescence can also contribute to higher levels of development and well-being in later periods of life (Guo, 2003).

This study highlights the crucial role of childhood parenting style in promoting positive adolescent and youth development, emphasizing the potential long-term benefits of nurturing and supportive parenting practices. Numerous studies have demonstrated a significant correlation between parenting style and “bile” (Feng et al., 2015). Furthermore, research has shown that self-reinforcement can strengthen this correlation (Liu et al., 2019), indicating that parenting style can not only directly affect bile, but also indirectly influence the strength of bile by impacting the self. In Traditional Chinese Medicine (TCM), “bile master decision” is believed to be essential, contributing not only to the body’s ability to defend against diseases, but also to the regulation of emotions, such as bravery, courage, fear, and the maintenance of mental and psychological health. Good bile levels are crucial for maintaining good interpersonal relationships (Li et al., 2015). Positive parenting practices have been shown to enhance self-acceptance levels, leading to healthier social relationships (Wang et al., 2021).

Human beings are social animals, and no one can exist without society. Interpersonal relationships are the channels that connect individuals with others and society, and they are also an important source of life significance (Zhang et al., 2020). Previous research has shown that the self plays an important role in the development of interpersonal relationships (Tang et al., 2015). For example, the interpersonal relationships of college students are significantly positively correlated with social self and psychological self (Wu and Wang, 2017). A longitudinal study has also demonstrated that the self at the first time point affects self-formation and interpersonal relationships at the second time point, and interpersonal relationships also affect self-development (Zhang et al., 2020). This is consistent with the findings of this study regarding the influence of adolescent self on adolescent interpersonal relationships.

Research by McKenzie has shown that the parent–child relationship in early family life affects later behavior patterns (Xu et al., 2008), and childhood parenting style has been found to affect interpersonal relationships in adolescence and young adulthood (Zhang et al., 2020). These findings are consistent with the results of this study on the influence of childhood parenting style on the interpersonal relationships of depressed youth.

This study validates and supports the importance of childhood parenting style on depressed patients, but parenting style is only one factor and definitely not the only factor. The scale responds to the patient’s own perceived parenting style, but depressed patients may also have a bias against their parents’ style, so it is helpful to correct the patient’s perception in therapy. If parenting styles do not make sense, and even some parents may be depressed and have difficulty supporting their children in times of distress, a combination of family therapy is often needed. Asian families are characterized by collectivism, interdependence, and familism (McCabe et al., 2020), a collectivist culture that tends to keep personal issues private within the family, and the fact that Chinese people tend to be facetious and have a morbid sense of shame about psychiatric problems, making it a barrier for people to seek treatment (Alvidrez et al., 2008). Research shows that Behavioral Parent Training (BPT) intervention can change parenting behaviors from authoritarian or permissive to authoritative to improve child externalizing behavior problems. Authoritative parenting (combined with high levels of warmth and control) can promote optimal child outcomes (McCabe et al., 2020). A brief intervention aimed at supporting parental need satisfaction reduced parental and child stress and improved parental adoption of need-supportive practices (Moè et al., 2020). For the patients themselves, gratitude, self-affirmation, goal setting, or meaningful things for seven consecutive weeks confirmed an increase in well-being and a decrease in ill-being for all groups (Moè, 2022). And a mobile intervention called Serene may help to reduce depressive symptoms through positive thinking, self-compassion and cognitive restructuring, which help to reduce over-identification with negative emotions (Al-Refae et al., 2021).

## Conclusion

Further analysis of the results suggests that the impact of childhood parenting style on adult depressed patients’ interpersonal relationships in the period of youth is mediated by three variables: adolescent self, courage, and the chain mediation of both variables. This study provides a retrospective cross-period influence development model to demonstrate that a stronger self and courage in adolescence can positively predict the development level of interpersonal relationships in the period of youth. Stronger self and courage levels in adolescence lead to better communication with others, less fear of rejection, and the ability to master different ways of getting along. These skills help establish better interpersonal relationships.

The chain mediating effect of adolescent self and courage on the relationship between childhood parenting style and depressive youth highlights how childhood parenting style influences the formation of an individual’s interpersonal skills and affects the interpersonal relationships in the period of youth through specific mediating variables in adolescence. This study sheds light on the internal mechanism of how childhood parenting style impacts the interpersonal relationships in the period of youth. In other words, childhood parenting style develops the interpersonal skills of adult depressed patients in the period of youth by influencing the formation of adolescent self and courage.

The study confirms the importance of childhood parenting style and suggests that this model can be used for the growth and development of depressed individuals. The model may be one of the growth and development of individuals with depression, but it reveals to us that when evaluating an individual’s development level in the current period, the influence of past personality factors should not be overlooked. By considering the current emotional reactions in the context of the personality formation process and “from past to present” systematically, it is possible to find more reasonable treatment ideas and methods.

## Data availability statement

The original contributions presented in the study are included in the article/supplementary material, further inquiries can be directed to the corresponding author.

## Author contributions

JC and KH collected and analyzed data together and wrote the manuscript. WW provided the article with guidance and modification. All authors contributed to the article and approved the submitted version.

## Funding

The research was supported by the National Natural Science Foundation of China, Grant No. 81603673, and by CI2021A03104 from Scientific and technological innovation project of China Academy of Chinese Medical Sciences.

## Conflict of interest

The authors declare that the research was conducted in the absence of any commercial or financial relationships that could be construed as a potential conflict of interest.

## Publisher’s note

All claims expressed in this article are solely those of the authors and do not necessarily represent those of their affiliated organizations, or those of the publisher, the editors and the reviewers. Any product that may be evaluated in this article, or claim that may be made by its manufacturer, is not guaranteed or endorsed by the publisher.
